# Client Profiles Among Medication Treatment Episodes for Opioid Use Disorder With and Without Co-occurring Mental Health Disorders

**DOI:** 10.21203/rs.3.rs-10656455/v1

**Published:** 2026-10-16

**Authors:** Daniel L. Howard, Kechen Zhao, Alanis Goncalves Leite, Yinfei Kong

## Abstract

Objectives To compare national medication treatment episodes for opioid use disorder with and without recorded co-occurring mental health disorders. Methods We analyzed 2023 US Treatment Episode Data Set-Discharges records for 109,115 publicly funded treatment episodes with methadone use at admission, known mental health comorbidity status, recorded sex, and discharge for completion, dropout, or facility termination. We compared demographics, substance use, treatment, and discharge characteristics. Results Episodes with co-occurring disorders (n = 56,403) more often involved female clients (40.2% vs 31.5%), White clients (67.4% vs 59.3%), injection drug use (37.1% vs 29.3%), alcohol use (26.0% vs 13.5%), cocaine or crack use (38.8% vs 20.3%), residential care (30.1% vs 11.4%), and prior treatment (85.4% vs 68.4%). Mean length of stay was shorter (128.0 vs 169.3 days). Conclusions Co-occurring mental health disorder status marked distinct client and service profiles. Public Health Implications: Opioid treatment programs should pair medication treatment with systematic mental health, polysubstance-use, and discharge-support planning.

